# Spectroscopy and Speciation Studies on the Interactions of Aluminum (III) with Ciprofloxacin and β-Nicotinamide Adenine Dinucleotide Phosphate in Aqueous Solutions

**DOI:** 10.3390/molecules17089379

**Published:** 2012-08-03

**Authors:** Xiaoling Ma, Li Li, Chongzheng Xu, Haiyan Wei, Xianlong Wang, Xiaodi Yang

**Affiliations:** 1 Nanjing Maternity and Child Health Care Hospital, Nanjing Medical University, Nanjing 210004, China; 2 Jiangsu Key Laboratory of New Power Batteries, School of Chemistry and Materials Science, Nanjing Normal University, Nanjing 210097, China; 3 Key Laboratory of NeuroInformation of the Ministry of Education, School of Life Science and Technology, University of Electronic Science and Technology of China, Chengdu 610054, China

**Keywords:** aluminum(III), ciprofloxacin (CIP), β-nicotinamide adenine dinucleotide phosphate (NADP)

## Abstract

In this study, both experimental and theoretical approaches, including absorption spectra, fluorescence emission spectra, ^1^H- and ^31^P-NMR, electrospray ionization mass spectrometry (ESI-MS), pH-potentiometry and theoretical approaches using the BEST & SPE computer programs were applied to study the competitive complexation between ciprofloxacin (CIP) and β-nicotinamide adenine dinucleotide phosphate (NADP) with aluminum (III) in aqueous solutions. Rank annihilation factor analysis (RAFA) was used to analyze the absorption and fluorescence emission spectra of the ligands, the binary complexes and the ternary complexes. It is found, at the μM total concentration level and pH = 7.0, the bidentate mononuclear species [Al(CIP)]^2+^ and [Al(NADP)] predominate in the aqueous solutions of the Al(III)-CIP and Al(III)-NADP systems, and the two complexes have similar conditional stability constants. However, the pH-potentiometry results show at the mM total concentration level and pH = 7.0, the ternary species [Al(CIP)(HNADP)] predominates in the ternary complex system. Comparing predicted NMR spectra with the experimental NMR results, it can be concluded that for the ternary complex, CIP binds to aluminum ion between the 3-carboxylic and 4-carbonyl groups, while the binding site of oxidized coenzyme II is through the oxygen of phosphate, which is linked to adenosine ribose, instead of pyrophosphate. The results also suggested CIP has the potential to be a probe molecular for the detection of NADP and the Al(III)-NADP complexes under physiological condition.

## 1. Introduction

**Figure 1:**
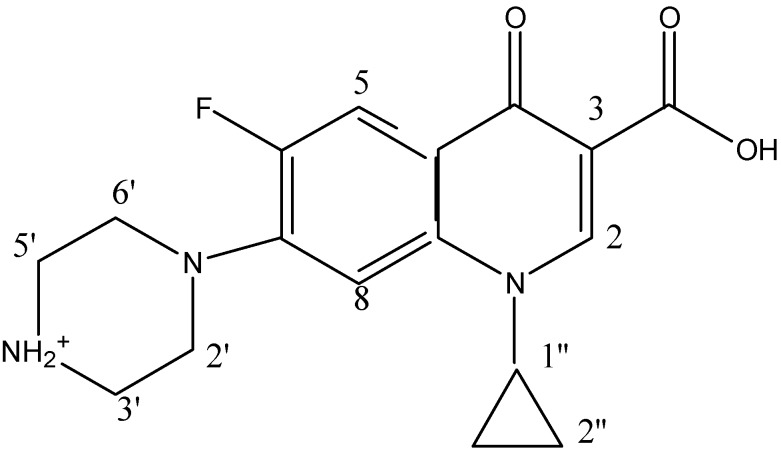
The structural formula of CIP.

Al has been found to affect the antioxidant role of some organs *in vivo*, which may be related to some aging diseases such as lipofuscin, Alzheimer’s and so on. Ciprofloxacin (CIP, Figure 1) is one of the most widely used second-generation members of the broad-spectrum fluoroquinolone antibacterial agent class. It can be persistently found among the top 200 most frequently prescribed drugs worldwide, with annual sales of more than $1 billion [1]. It has been found that fluoroquinolone drugs show strong binding ability towards Al(III) [2]. The co-administration of CIP and other fluoroquinolone antimicrobials such as levofloxacin and lomefloxacin with Al(III)-containing anti-acid agents could lower the adsorptivity of the drugs due to the chelating effect of the metal. On the other hand, large amounts of fluoroquinolone drugs have been administered to both human beings and animals. Depending on the chemical nature of the drugs and the physiological processes involved, the drugs are excreted as the parent compounds, as conjugates, or as oxidation or hydrolysis products of the parent compounds. Therefore, a considerable portion of the compounds can reach the soil environment and represent a potential contamination risk to plants and animals [3,4,5,6,7,8]. Recent studies have found that the sorption of CIP to hydrous oxides of Al(III) on the soil surface through a mononuclear complexation is very strong [3,4,5,7]. Therefore, the determination of the structure is of great significance for further research of the ecological problem of ciprofloxacin residues in soil, and other issues of pharmacological interactions and fluorescence analysis methods.

**Figure 2:**
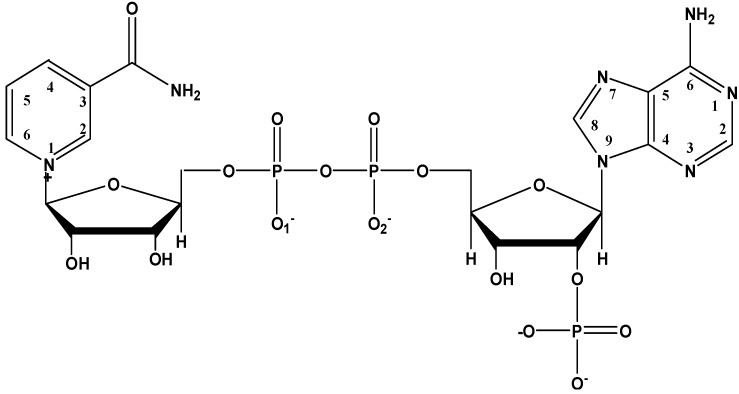
The structure of NADP.

Nicotinamide adenine dinucleotide phosphate (NADP, Figure 2) and its reduced form NADPH are important coenzymes in both plants and animals [9]. Like other phosphate molecules of biological importance, such as adenosine monophosphate (AMP), diphosphate (ADP) and triphosphate (ATP), NADP shows strong binding capability with Al(III) via the complexation of the nonbridging phosphate group [10,11,12,13]. Aluminum can cause oxidative damage to cellular biological processes by inhibiting glutathione regeneration through the inhibition of NADPH supply in mitochondria [10]. How do such natural compounds affect the binding and adsorption of fluoroquinolone drugs in the presence of relative high Al(III) concentration? In our studies on the interactions between Al(III) and small molecules of biological interests [14,15,16,17,18], we investigated the competitive complexation of Al(III) with these two biological ligands under physiological conditions through spectroscopic and electronic methods. We found that the complexation of Al(III) with CIP causes a red-shift of the fluorescence emission spectra and an increase in intensity. In our former research and some of the literature [18,19], it is proposed that CIP possibly binds to aluminum ion between the 3-carboxylic and 4-carbonyl groups, while the binding site of NADP was the oxygen of phosphate, which was linked to adenosine ribose, instead of pyrophosphate. Since NADP participates in basal metabolism with various zymoproteins in the form of holoenzymes, the change will affect the metabolic pathway. The study of interaction between NADP and CIP with Al(III) has great significance to further reveal the detailed molecular-level information about the coordination and the structure of the complexes under physiological conditions.

## 2. Result and Discussion

### 2.1. Electronic Spectra of CIP and NADP and Their Complexes with Al(III)

**Figure 3:**
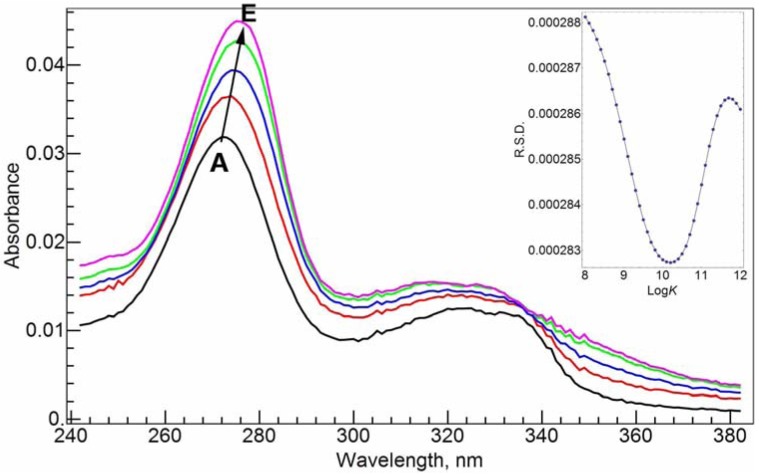
Absorption spectra of CIP and Al(III)-CIP at different molar ratios of metal to ligand. The inset shows the curve of *RSD* *vs*. log*K* for the RAFA analysis of the spectra. **A**–**E**: 20 µM *c*_CIP_, 10 mM Tris-HCl buffer solution (pH = 7.0); *c*_Al(III)_, 0 (**A**), 5 (**B**), 10 (**C**), 15 (**D**) and 20 (**E**) µM.

In Tris-HCl buffer solution at pH = 7.0, the absorption spectrum of 20 µM CIP has a strong absorbance band at 272.0 nm and two much weaker absorbances near 323.0 nm (error: ±0.1 nm, the subsequent errors are the same) and 335.0 nm, respectively (Figure 3A). Upon complexation with Al(III), the major absorbance band at 272.0 nm shifts slightly to the red direction (Figure 3C–E). After the addition of 1 molar equivalent of Al(III), the maximum absorbance peak shifts to 275.0 nm. The red shift suggests that the complexation between Al(III) and CIP via the carboxylate and ketone groups might form a six-member ring, and disturb the conjugation system of the fluoroquinolone structure.

**Figure 4:**
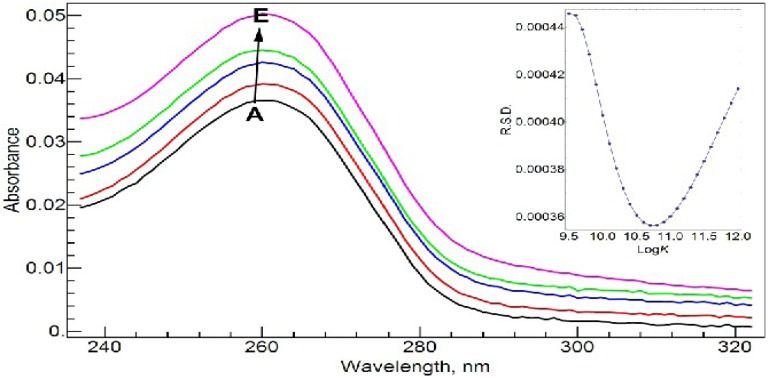
Absorption spectra of NADP and Al(III)-NADP at different molar ratios of metal to ligand. The inset shows the curve of *RSD* *vs*. log*K* for the RAFA analysis of the spectra. **A**–**E**: 20 µM *c*_NADP_, 10 mM Tris-HCl buffer solution (pH = 7.0); *c*_Al(III)_, 0 (**A**), 5 (**B**), 10 (**C**), 15(**D**) and 20 (**E**) µM.

Figure 4 shows the absorption spectra of 20 µM NADP and its complex with Al(III) in Tris-HCl buffer solution at pH = 7.0 at different molar ratios of metal to ligand. NADP has an absorbance band at 261.0 nm due to the adenine ring. As well known, Al(III) shows strong binding ability with the phosphate group in NADP [20,21]. The complexation does not cause a change in the maximum absorbance wavelength, but the absorbance intensity increases with the concentration of Al(III) in a nearly linear fashion.

**Figure 5:**
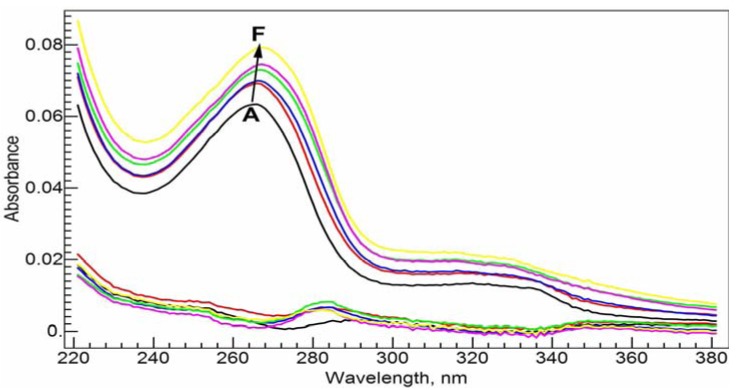
Absorption spectra of the ternary Al(III)-CIP-NADP system at different molar ratios of metal to ligand. The bottom curves are the calculated residual spectra using the RAFA analysis. **A**–**F**: 20µM *c*_NADP_, 20µM *c*_CIP_, 10 mM Tris-HCl buffer solution (pH = 7.0); *c*_Al(III)_, 0 (**A**), 5 (**B**), 10 (**C**), 15 (**D**), 20 (**E**) and 25 (**F**) µM.

In Figure 5, we first mixed 20 µM NADP and 20 µM CIP in Tris-HCl buffer solution at pH = 7.0 and then measured how the absorption spectra of the ligands change upon the addition of Al(III) at different concentrations. Because the major absorbance bands of CIP and NADP overlap to a great degree, not much new information appears at first sight. We decided to calculate the conditional stability constants of Al(III) with the two ligands from the spectra in Figure 3 and Figure 4. Without considering the protonation states of the ligands, the complexation reactions can be written as the following equations, where *K_a_* and *K_b_* are the corresponding complex formation constants and charges are ignored for the ligands and the complexes: 


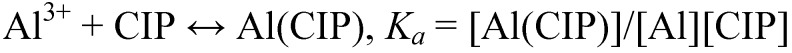
(1)


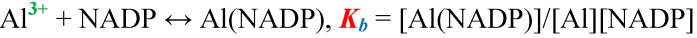
(2)

At pH = 7.0, we need to take the hydrolysis reactions of Al(III) into account. The reactions can be generally written as follows:


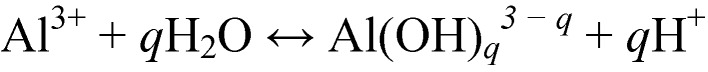
(3)

The accumulated hydrolysis constants in the common logarithm (lg*β*) for *q* = 1, 2, 3 and 4 are −5.5, −11.1, −16.6 and −23.2 (25 °C, *I* = 0.16), respectively, for producing the Al(OH)^2+^, Al(OH)_2_^+^, Al(OH)_3_ and Al(OH)_4_^−^ species.

Rank annihilation factor analysis (RAFA) has been a well-studied and effective method to calculate the stability constants from spectroscopic data [22,23,24,25,26]. The method merits from using all the range of spectroscopic intensity values at different molar ratios instead of using just the maximum peak absorbance values. In addition there is no need to know the spectrum of the complex in advance. The whole-range spectroscopic data at a series of molar ratios form a matrix, while the spectrum of the pure ligand is used to decrease the rank of the matrix by 1. Principal component analysis (PCA) is used to analyze the matrix. The eigenvalues of the covariance matrix of the spectroscopic data are used to determine the major species contributing to the spectra. The commonly used relative standard deviation (RSD), which was calculated from the eigenvalues, was adopted to find the stability constants. See the insets of Figure 3 and Figure 4.

Through the RAFA method, we found that the spectra in both Figure 3 and Figure 4 are mainly due to two species, the ligand and 1:1 complex. The possibility for the 1:2 (M/L) complex is small. The conditional stability constants at pH = 7.0 are found to be log*K_a_* = 10.2 and log*K_b_* = 10.7. NADP shows slightly stronger binding ability with Al(III) than CIP. With these two conditional stability constants and the following assumptions: (1) the interaction between the ligands (CIP and NADP) is negligible, and (2) at the μM concentration level, the chance to form the mixed ternary complex, Al(CIP)(NADP) is small, we calculated the synthetic spectra at the conditions in Figure 5 from the spectra of the pure ligands and the extrapolated spectra of the ternary complexes. The residual spectra after subtracting the synthetic spectra from the corresponding real spectra are shown in the bottom of Figure 5. As shown, the residual spectra are largely identical except for a small region near 290.0 nm. This result can be interpreted as meaning that the above assumptions are largely sound, and the residual spectra represent a consistent system effect from the interaction between CIP and NADP.

### 2.2. Effect of NADP on Fluorescence Spectra of CIP and Its Complex with Al(III)

**Figure 6:**
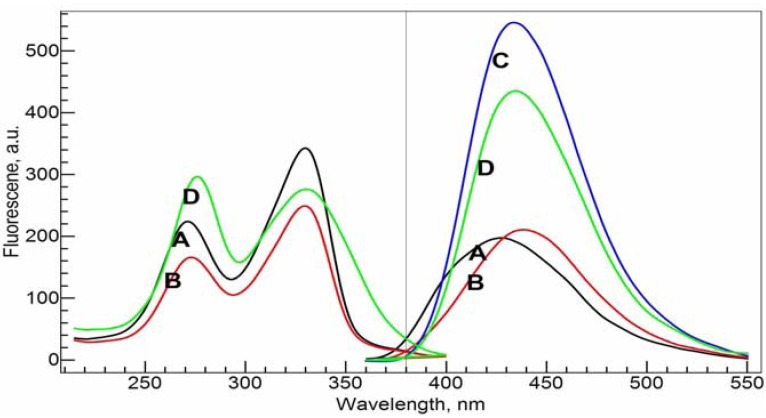
Fluorescence excitation (left panel) and emission (right panel) spectra of the ligands and the complexes. **A**–**D**: 10 mM Tris-HCl buffer solution (pH = 7.0); **A**, 34 μM *c*_CIP_ only; **B**, 34 µM *c*_CIP_ and 20 µM *c*_NADP_; **C**, 34 µM *c*_CIP_ and 20 µM *c*_Al(III)_; **D**, 34 μM *c*_CIP_, 20 µM *c*_NADP_ and 20 µM *c*_Al(III)_. Excitation wavelength was fixed at 270 nm forthe emission spectra and emission wavelength was fixed at 430 nm for the excitation spectra.

Fluoroquinolones are a kind of compound with strong fluorescence, while NADP is transparent in fluorescence. Therefore, fluorescence spectroscopy fits well the purpose to study how NADP affects the complexation between Al(III) and CIP. In Figure 6, the emission and excitation spectra of CIP alone (A), CIP with NADP (B), Al(III) with CIP (C), and Al(III) with CIP and NADP (D) in Tris-HCl buffer solutions at pH = 7.0 are shown. Consistent with the UV-visible absorbance spectra, the ligand CIP shows two strong peaks in the excitation spectra at 271.0 nm and 330.0 nm. The former wavelength was chosen as the excitation wavelength for the following emission spectra experiments. The ternary system of Al(III)-CIP-NADP, the excitation peak at 271.0 nm shifts to 275.0 nm.

The ligand CIP itself has a strong emission band with a maximum emission wavelength at 426.0 nm. Upon the addition of NADP, this peak shifts to 438.0 nm while the emission intensity stays the same. This indicates that there is indeed an interaction between the two ligands. With the addition of Al(III), the emission intensity increases significantly, to about 2.5 times that of the original ligand. Furthermore, the complexation also causes a red-shift of the emission spectra. The maximum emission peak of the Al(III)-CIP complex system is at 433.0 nm. With the addition of one molar equivalent of NADP [relative to Al(III)] to the binary Al(III)-CIP system, the peak decreases its intensity significantly, by about 20% of the intensity of the Al(III)-CIP complex system. On the other hand, the maximum emission wavelength shifts slightly to 435.0 nm. Qualitatively, the results show us that NADP has strong binding ability with Al(III) compared with CIP. The formation of Al(NADP) complex decreases the concentration of Al(CIP) complex and causes the intensity decreases for the complex.

The Al(CIP) complex formation and/or the competition between CIP and NADP of Al(III) seem strongly dependent on pH, and around the neutral condition of pH = 7 the emission spectrum of the complex system reaches to its maximum magnitude (data not shown).

**Figure 7:**
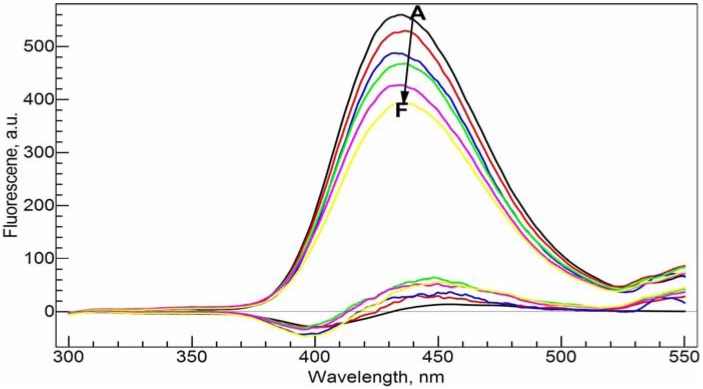
Fluorescence emission spectra of the ternary Al(III)-CIP-NADP system at different molar ratios of metal to ligand. The bottom curves are the calculated residual spectra using the RAFA analysis. **A**–**F**: 34 µM *c*_NADP_, 20 µM *c*_CIP_, 10 mM Tris-HCl buffer solution (pH = 7.0); *c*_Al(III)_, 0 (**A**), 5 (**B**), 10 (**C**), 15 (**D**), 20 (**E**) and 25 (**F**) µM. Excitation wavelength was 270 nm.

In Figure 7, NADP was gradually added to the Al(III)-CIP system. The emission intensity decreases in a nearly linear fashion with the increase of NADP concentration. We also calculated the residual spectra as the difference between the real spectra and the synthetic spectra based on the speciation distribution calculated from the conditional stability constants obtained from the previous section. The residual spectra have a negative peak around 395.0 nm and a positive peak around 450.0 nm. Both are increasing with the concentration of NADP. This is consistent with the red shift effect caused by the interaction between NADP and CIP.

**Figure 8:**
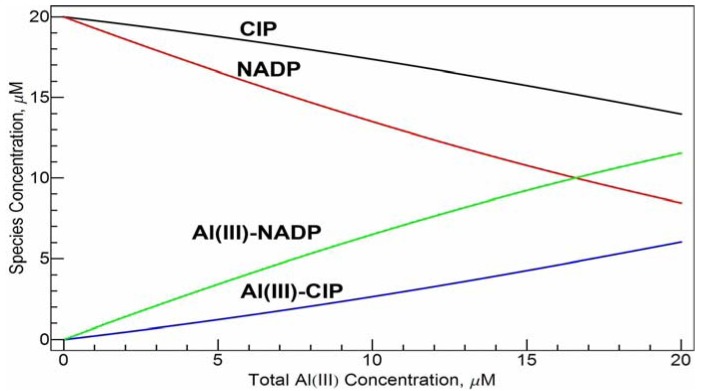
Speciation distribution of the system of 20 µM *c*_CIP_ and 20 µM *c*_NADP_*vs*. *c*_Al(III)_ concentration at pH =7.0 calculated with the conditional stability constants obtained in this work. CIP and NADP represent the free ligands and Al(III)-CIP and Al(III)-NADP represent the mononuclear complexes.

The emission spectra were also taken with the increasing Al(III) concentration while keeping the concentrations of NADP and CIP constant at 20 µM in Tris-HCl buffer solution at pH = 7.0 and the increasing concentration of CIP while keeping the concentrations of NADP and Al(III) constant at 20 µM in Tris-HCl buffer solution at pH = 7.0 (spectra not shown). The intensity of the emission band shows a nearly perfect correlation with the concentration of Al(III) or CIP. This is not surprising. Since the conditional stability constants of the two complexes are close, the species distribution of the free ligands and the complexes has an approximately linear dependence on the total concentration of Al(III) (see Figure 8). Such a linear relationship may be used in quantitative determination of Al(III) and/or the ligands under physiological conditions.

### 2.3. Coordination Modes Identified with ^1^H and ^31^P-NMR Spectra

**Figure 9:**
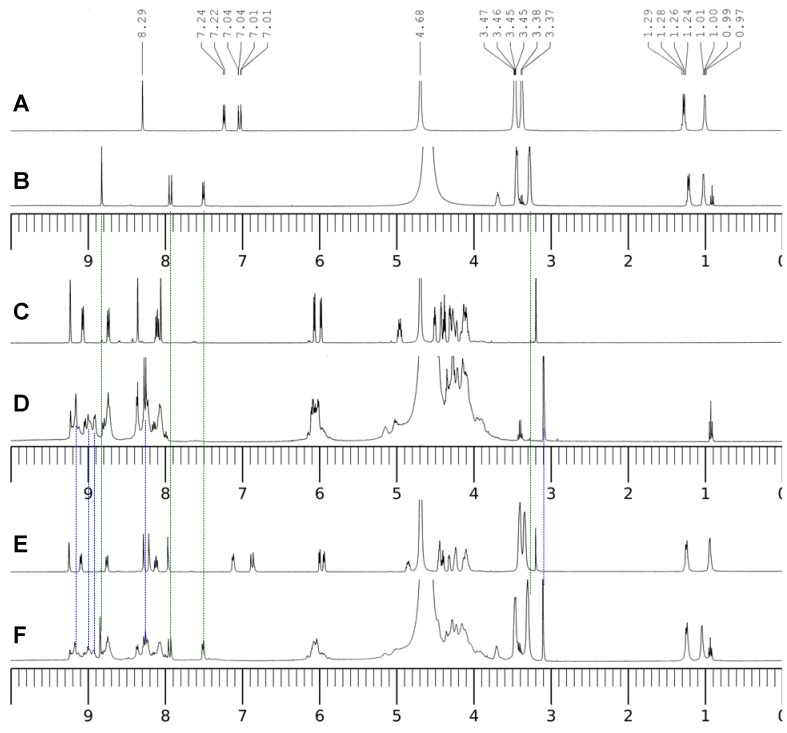
^1^H-NMR spectra of the ligands and the complexes. **A**–**F**: pD = 3.0; **A**, 10 mM *c*_CIP_ only; **B**, 10 mM *c*_CIP_ and 10 mM *c*_Al(III)_; **C**, 10 mM *c*_NADP_ only; **D**, 10 mM *c*_NADP_ and 10 mM *c*_Al(III)_; **E**, 10 mM *c*_CIP_ and 10 mM *c*_NADP_; F, 10 mM *c*_CIP_, 10 mM *c*_NADP_ and 10 mM *c*_Al(III)_.

To identify the coordination modes between the ligands and Al(III), ^1^H-NMR spectra were measured for the D_2_O solutions of the ligands, the binary complex systems and the ternary complex system at pD = 3.0 (see Figure 9). This acidic pH value was chosen to avoid the possible hydrolysis of Al(III) under the neutral pH conditions at the much elevated concentration of 0.10 M. Based on the ^1^H-NMR evidence, Sakai *et al.* concluded CIP along other fluoroquinolone antimicrobials form stable complexes with Al(III) at pD = 2.5 and the absorptivity of the drugs in oral administration could be affected by Al(III) [2]. Compared with CIP, the complex Al(CIP) shows a down-field shift for the three aromatic protons on the quinolone ring. The proton on C-2 shifts from 8.29 to 8.81 ppm, the doublet peaks of the protons on C-5 and C-8 shift from 7.02 and 7.23 ppm to 7.50 and 7.92 ppm, respectively. The hydrogens on the piperazine at 3.46 and 3.38 ppm in CIP shift to upfield by only 0.02 ppm. The download shifts are caused by the induction effect due to the strong coordination of Al(III) with the 4-keto group and the 3-carboxylate group by forming a 6-member ring. Since the signals from the ligand itself disappeared completely, the Al(III)-CIP complex was the main species in the solutions. Using the ^1^H-NMR titration experiments, Sakai *et al*. estimated that the activation free energy for the dissociation of the Al(III)-CIP complex is *ca.* 19 kcal/mol pD = 2.5 [2]. This value is close to the Gibbs free energy for the dissociation reaction, 14 kcal/mol, calculated from the conditional stability constant in the UV-visible spectra experiments at pH = 7.0. Sakai also proposed that besides the main M/L = 1:1 species Al(CIP), there also exist the 1:2 and 1:3 species, Al(CIP)_2_ and Al(CIP)_3_ at the very high concentration of Al(III) and CIP [2]. We could not exclude the possibility for the presence of Al(CIP)_2_ and Al(CIP)_3_ in the ^1^H-NMR experiments, but their percentage at the very low concentration (μM) level under physiological conditions should be very low.

**Figure 10:**
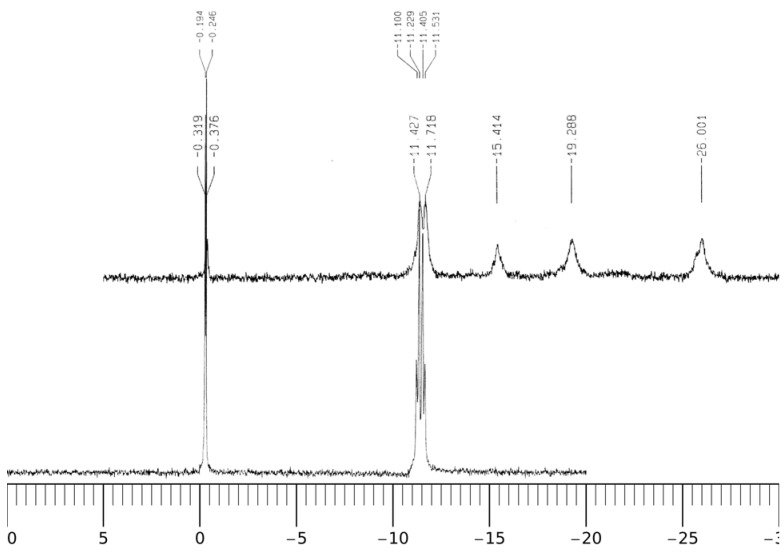
^31^P-NMR spectra of NADP and Al(III)-NADP. **A**–**B**: pD = 3.0; **A**, 10 mM *c*_NADP _only; **B**, 10 mM *c*_NADP_ and 10 mM *c*_Al(III)_.

Figure 9C,D and 10 show the ^1^H-NMR and ^31^P-NMR spectra of NADP and the Al(III)-NADP complex system at pD = 3.0. In the free ligand of NADP, the proton signals on both the nicotinamide and adenine ring appear in the region between 8.0 and 9.3 ppm and overlap with each other. Upon the addition of one molar equivalent of Al(III), the proton resonance signals become quite complicated due to three kinds of changes: (1) the broadening effect because of the scalar coupling of the quadrupolar aluminum ion; (2) the upfield shifts, which was thought due to the alterations in geometry and dielectric constant of the solvent environment; (3) the separation of the nicotinamide and adenine proton signals upon the coordination of Al(III) with the nonbridging phosphate group. Besides the upfield shifts of the aromatic hydrogens, the proton signals at 6.0 ppm, which were assigned to the hydrogens on the carbon atoms directly connected with the phosphate groups on the two ribose rings, become separated because the nonbridging phosphate binds with Al(III) and causes the downfield shift of one hydrogen. In addition, ribose hydrogen at 3.18 ppm in the free ligand shifts slightly to 3.10 ppm in the complex.

In the ^31^P-NMR spectra in Figure 10, the phosphorus resonance signal appears at 11.3 ppm in NADP. In the binary Al(III)-NADP complex system, three new signals appear in the upper field at −15.4, −19.3 and −26.0 ppm. The phenomenon is similar to the findings by Karlik *et al*. [27]. The complexation of Al(III) with glucose-6-phosphate also causes a similar upfield shift pattern at −12.9, −16.8 and 19.0 ppm [22]. This result indicates there are least three complexation configurations.

Figure 9 also shows the ^1^H-NMR spectra of the binary system CIP and NADP (E) and the ternary system Al(III)-CIP-NADP (F). In the binary system, the hydrogens on the adenine –NH_2_ group shift upfield from 8.09 ppm to 7.96 ppm. This may be due to the hydrogen bonding between the –NH_2_ group and the –COOH group on CIP. Such interaction also causes the red shift of the UV-visible and fluorescence spectra. The spectrum of the ternary system shows the characteristic peaks of both the Al(III)-CIP complex and the Al(III)-NADP complex and may be seen as a Al(III)-CIP-NADP complex. 

### 2.4. Electrospray Ionization Mass Spectrometric Studies and Computational Modeling

Electrospray ionization mass spectrometry (ESI-MS) was used to characterize the species in the complex systems. 11A, B shows the spectra of the Al(III)-CIP complex solution in Tris-HCl buffer solution at pH = 7.0, measured in the negative and positive ion modes, respectively. In spectrum A, the complex [Al(CIP)(H_2_O)_5_]^−^ species appears near *m/z* = 448. A series of de-hydrogenated molecule products appear at *m/z* = 446, 444, 442, 440 and 438. In spectrum B, the *m/z* = 161 peak can be assigned to the decomposed complex species, [Al(COO)(H_2_O)_5_]^+^.

**Figure 11:**
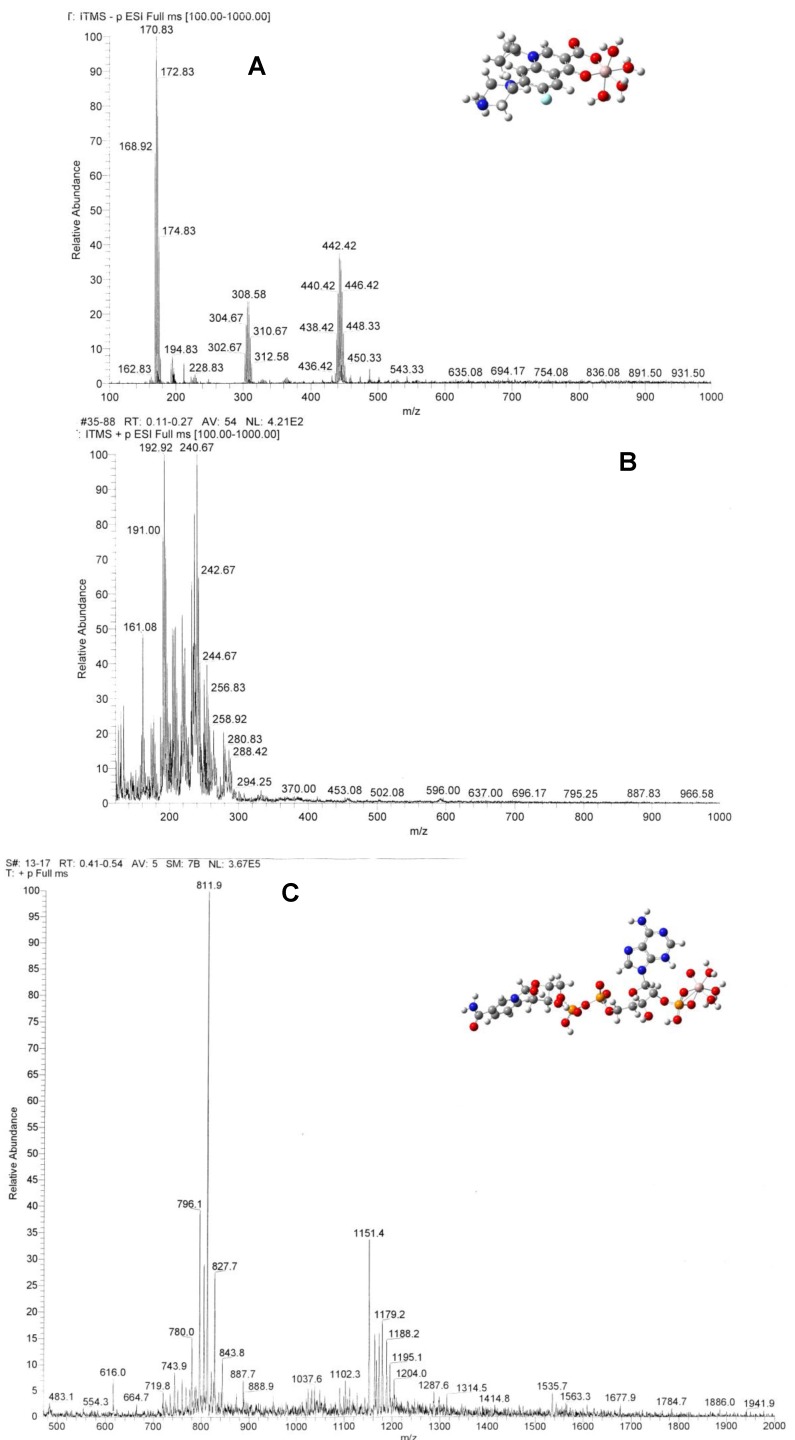
(*Color online*) Electrospray ionization mass spectra of Al(III)-CIP in negative ion mode (A) in positive ion mode (**B**) and of Al(III)-NADP (**C**). The insets of panels A and C showed the optimized structure of the complexes at the B3LYP/6-31G(d)//B3LYP/6-31G(d) level. Color codes: C, dark gray; H, light gray; O, red; N, blue; P, orange; Al, purple.

Figure 11C shows the spectrum of the Al(III)-NADP complex system measured in the positive ion mode in Tris-HCl buffer solution at pH =7.0. The major peak at *m/z* = 812 is due to the ligand adduct with three Na ions, [Na_3_NADP]^+^. The molecular peak of NADP^+^ appears at *m/z* = 744. The *m/z* = 616 peak is due to a loss of the nicotinamide group from the ligand. The *m/z* = 804 peak can be assigned to the complex species, [Al(H_2_O)_5_(NaNADP)]^+^. There is no peak representing the M/L = 1:2 complexes.

The insets of Figure 11A,C show the optimized geometry structure of the complexes in vacuum with the B3LYP/6-31G(d) level of theory. It is found that Al(III) can strongly bind with the keto and carboxylate groups of CIP forming a six-member ring. The electronic structure of the quinolone ring is slightly disturbed, which causes the spectroscopic changes between the ligand and the complexes. Al(III) binds with NADP through the complexation with the nonbridging phosphate group in a bidentate configuration. Although aluminium ion is not directly connected to the adenine ring, it is found that the complexation causes the adenine ring to turn about 90 deg around the C-N bond. Such conformational change would change the superconjugation effect between the aromatic adenine and the ribose ring and explain the spectroscopic changes of Al(III)-NADP complex compared with the ligand itself.

### 2.5. Speciation on the Ternary Al(III)-CIP- NADP Systems

**Figure 12:**
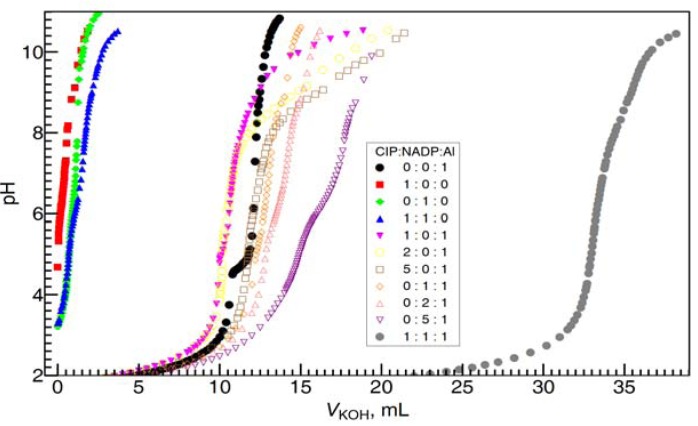
Titration data points measured in the Al(III)-NADP-CIP complex systems (C_Al_ = 0.001 M, C_KOH_ = 0. 1026 M) at various metal ion to ligand ratios in 0.20 M KCl medium at 25 °C The fitted formation constants of the complexes are given in Table 1.

In order to investigate the possibility formation of mixed-ligand complexes, especially, the Al(III)-CIP-NADP ternary systems, and the complexation species formation constants, pH-potentiometric titrations and computational modeling of the mixed-ligand complexes were performed in aqueous solution. In Figure 12, the pH-potentiometric titration of different various metal ion to ligand ratios of Al, CIP and NADP were well carried out from pH = 2 to pH = 11. Our macroscopic constants (Table 1) are in accordance with those obtained by other authors [24]. These values are in agreement with those reported in the literature for the same molecular. The small difference of this work and literature [25] may due to the corresponding thermodynamic data obtained at different ionic strength. The results of potentiometric data of the complexation equilibriums between NADP, CIP and Al are shown in Table 1.

**Table 1:** Proton and Al(III) stability constants for CIP and NADP binary and ternary complexes at 25 °C and *I* = 0.20 M (KCl) *^a^*.

Complex species	CIP		NADP		CIP and NADP
	This work	Reference [24]	This work	Reference [25]	This work
*Protonation*					
HL	8.60(0.03)	8.54	9.93(0.03)	11.50	
H_2_L	14.76(0.03)	14.61	16.09(0.04)	16.44	
H_3_L			19.90(0.06)	20.19	
H_4_L				21.39	
*Binary complexes*					
AlL	9.36(0.03)		10.71(0.02)		
Al(HL)	15.85(0.08)		16.01(0.01)		
Al(H_2_L)			19.16(0.04)		
AlL(HL)	21.38(0.03)				
Al(H_2_L)_2_			37.84(0.06)		
AlL_2_(HL)			29.82(0.02)		
Al(H_2_L)_3_			57.53(0.04)		
*Ternary complexes*					
Al(CIP)(HNADP)					26.56(0.02)
Al(HCIP)(HNADP)					32.49(0.02)

*^a^* Standard deviations are given in parentheses.

**Figure 13:**
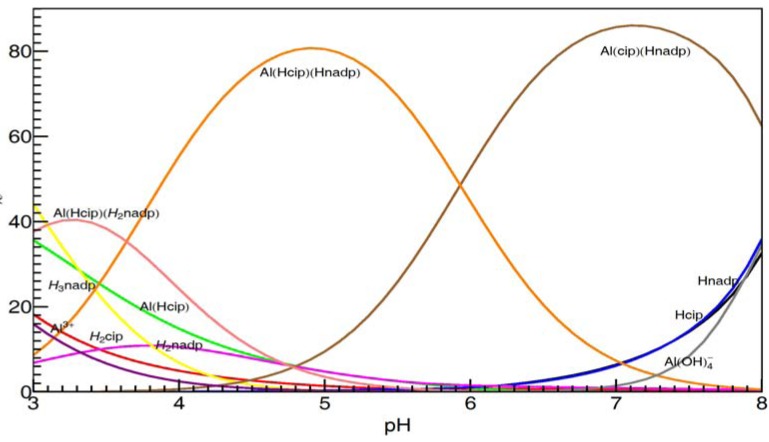
Species distribution curves for the complexes formed in the Al(III)-NADP-CIP complex system, at 25 °C, 0.2 M KCl and the concentration of Al is 10 mM in the case of Al: NADP:CIP = 1:1:1 system.

Also, Figure 13 shows the calculated distribution diagram of Al with CIP and NADP solutions, from pH 3.0 to pH 8.0, 25 °C 0.2 M KCl, at the concentration of Al is 10 mM in the case of Al: NADP:CIP = 1:1:1 system.

Different with former spectral studies, our pH-potentiometric studies show, in the Al(III)-CIP-NADP ternary systems, at mM concentration level, the 1:1:1 ternary complexes are the predominant species in acidic aqueous solution. At pH 3.0, the 1:1:1 complex [Al(HCIP)(H_2_NADP)] is the predominant species, while, at pH 5.0, the other 1:1:1 complex [Al(HCIP)(HNADP)] becomes the major species. Finally, at around neutral pH, the 1:1:1 complex [Al(CIP)(HNADP)], in which the CIP is deprotonated, became the major species under physiological pH. These results are proved by the ^1^H-NMR spectra above.

## 3. Experimental

### 3.1. Chemicals

CIP and NADP stock solutions (1.00 mM) were prepared, respectively, by dissolving CIP hydrochloride salt (USP Grade, Hefei Bomei Biotechnology Co. Ltd., Hefei, China) and NADP disodium trihydrate salt powder (>98.0%, Bio Basic Inc., Markham, Canada) with double-distilled water, and stored at 4 °C. Tris-HCl buffer solution (0.10 M, pH = 7.0) was prepared by dissolving tris(hydroxymethyl)aminomethane (analytical regent grade, Sinophram Chemical Reagent Co. Ltd., Ningbo, China) with double-distilled water, and the pH was adjusted with hydrochloric acid. Al powder (≥99%, Sinopharm Chemical Regent Co. Ltd.) was dissolved with hydrochloric acid (analytical regent grade, Sinophram Chemical Reagent Co. Ltd.) to prepare Al(III) stock solution (10.00 mM, pH = 1.70). D_2_O solvent and AlCl_3_ (≥98%, Sinopharm Chemical Regent Co. Ltd.) were used to prepare the solutions for the ^1^H-NMR, and ^31^P-NMR experiments.

### 3.2. Spectroscopic Measurements

Mixed solutions were prepared from the stock solutions to the designated concentrations. Before taking spectroscopic measurements, the solutions were shook well and let stand on table for 20 min at room temperature (*ca.* 298 K). Fluorescence spectra were taken with a Model LS50B Fluorescence Spectrophotometer (Perkin-Elmer, Massachusetts, MA, USA) and absorption spectra were taken with a Cary 5000 UV-Vis-NIR Spectrophotometer (Varian associates inc., California, CA, USA).

^1^H-NMR measurements were performed at room temperature (298 K) on a Bruker AVANCE 400 spectrometer (Bruker AXS GmbH, Karlsruhe, Germany) operated at 400 MHz. Chemical shifts were referenced to D_2_O (4.70 ppm) for 1H-NMR spectra and 32 scans were accumulated for each experiment. ^31^P-NMR spectra were taken at room temperature (298 K) on a Bruker DRX500 spectrometer operated at 201.5 MHz. Chemical shifts were referenced to a coaxial insert containing 0.1 M H_3_PO_4_ (0 ppm) in every sample.

Electrospray ionization mass spectrometry (ESI-MS) experiments were performed in positive-ion and negative-ion modes with a Finnigan Mat LCQ mass spectrometer (Thermo Finnigan, San Jose, CA, USA). The scan range was from 500 *m/z* to 2000 *m/z* for the Al-NADP system, and from 100 *m/z* to 1000 *m/z* for the Al-CPFX system.

### 3.3. Stability Constant Calculations

Rank annihilation factor analysis (RAFA) was used to calculate the conditional stability constants between Al(III) and the ligands from the spectrophotometric data at different metal/ligand molar ratios. The relative standard deviation (RSD) measure in principal component analysis (PCA) was used to determine the number of principal species in the complexes, and to find the conditional stability constants at pH = 7.0. It was calculated from the eigenvalues of the covariance square matrix of the spectroscopic absorbance or intensity data. Detailed explanation on the theory of RAFA can be found in the literature [22,23,24,25,26]. The method is implemented in Mathematica (Wolfram Research Inc., Champaign, USA) and the code is available upon request from the authors.

The stability constants of the ligand and main metal complexes are calculated with the aid of the computer program SPE & BEST [14] in the pH-metric measurements. The logarithmic value of the ionic product of water was found to −13.78 at 25 °C in 0.2 M KCl medium [28]. The formation of hydroxo complexes of Al(III) was also taken into account in the calculations, which were shown in Table 1.

### 3.4. pH-Metric Measurements

The stability constants of the proton and Al complexes of the ligands (CIP and NADP) were determined by pH-metric titrations of 50.0 mL samples. The concentration of the ligand was 0.001M, and the metal ion to ligand ratios were 0:1, 1:1, 1:2, 1:5 for the binary system and 1:1:1 for the ternary system, respectively. The potentiometric measurements were conducted at 25 °C in 0.2 M KCl ionic medium. The temperature was maintained at 25.0 ± 0.1 °C by circulating the thermostated water through the jacket. Duplicate titrations were carried out and the reproducibility of the titration curves was within 0.01-pH unit throughout the whole pH range. Because of the rather sluggish ligand-exchange kinetics of Al and the precipitation reactions, when equilibration could not be reached in 10 min, the corresponding titration points were omitted from the calculations [14]. Complexes were added in the model one at a time until the value of lowest σ_fit_ was achieved (usually less than 0.01). The pH value was measured with the Seven Multi-pH meter (Mettler-Toledo Instruments Co., Ltd., Shanghai, China) with a glass combination electrode, which was firstly calibrated for hydrogen ion concentration according Irving *et al*. [14,15].

### 3.5. Electronic Structure Calculations

Complexation modes with Al(III) of CIP and NADP were also modeled by calculations of electronic structures. The starting point structures of CIP and CIP-Al(III) complex were built based on the X-ray crystallographic structures of CIP and CIP-Cu(II) complexes from the Cambridge Crystal Structural Database (reference codes were QEKGAP and QELJW) [26]. The starting point structure of NADP was built from the X-ray crystallographic structure of human aldose reductase complex with NADP and the inhibitor IDD594 from the Protein Data Bank (reference code is 1US0) [29,30].

Geometry optimization and energy calculation were performed on the isolated molecules of the ligands and the complexes with the hybrid density functional theory, B3LYP/6-31G(d)//B3LYP/6-31G(d) model chemistry [31]. Normal mode analysis was used to confirm the optimized structures were indeed the energy minima. All the electronic structure calculations were carried out with the Gaussian 03 suite [27].

## 4. Conclusions

From the above results and discussion, the following conclusions can be drawn:

(1) In neutral pH aqueous solutions, both CIP and NADP are strong binders of Al(III) with comparable binding ability.
(2) At µM concentration level and in neutral pH aqueous solutions, the 1:1 species, *i.e.*, [Al(CIP)]^2+^ and [Al(NADP)], predominate. However, according to our pH-potentiometric studies, at mM concentration levels and neutral pH values, the 1:1:1 species, *i.e.*, [Al(CIP)(HNADP)], becomes the major species.
(3) The conditional stability constants of [Al(CIP)]^2+^ and Al(NADP) at μM concentration level near pH = 7 are 10^10.2^ and 10^10.7^, respectively. In the binary and ternary systems, and many other complexation species formation constants were obtained by pH-potentiometric studies.
(4) Fluorescence of CIP in neutral aqueous solutions was promoted upon complexation with Al(III). Due to the competitive coordination from NADP, addition of NADP to the Al(III)-CIP complex solutions cause the fluorescence decrease. In certain ranges, the magnitude of the intensity decrease shows linear correlation with the concentration of NADP. Thus, CIP has the potential to be an analytical agent for the detection of NADP and the Al(III)- complex under physiological condition.

## References

[1] Mitscher L.A. (2005). Bacterial topoisomerase inhibitors: Quinolone and pyridone antibacterial agents. Chem. Rev..

[2] Sakai M., Hara A., Anjo S., Nakamura M. (1999). Comparison of the complexation of fluoroquinolone antimicrobials with metal ions by nuclear magnetic resonance spectroscopy. J. Pharm. Biomed. Anal..

[3] Carrasquillo A.J., Bruland G.L., MacKay A.A., Vasudevan D. (2008). Sorption of ciprofloxacin and oxytetracycline zwitterions to soils and soil minerals: Influence of compound structure. Environ. Sci. Technol..

[4] Gu C., Karthikeyan K.G. (2005). Sorption of the antimicrobial ciprofloxacin to aluminum and iron hydrous oxides. Environ. Sci. Technol..

[5] MacKay A.A., Seremet D.E. (2008). Probe compounds to quantify cation exchange and complexation interactions of ciprofloxacin with soils. Environ. Sci. Technol..

[6] Tolls J. (2001). Sorption of veterinary pharmaceuticals in soils: A review. Environ. Sci. Technol..

[7] Trivedi P., Vasudevan D. (2007). Spectroscopic investigation of ciproﬂoxacin speciation at the goethite- water interface. Environ. Sci. Technol..

[8] Golet E.M., Strehler A., Alder A.C., Giger W. (2002). Determination of fluoroquinolone antibacterial agents in sewage sludge and sludge-treated soil using accelerated solvent extraction followed by solid-phase extraction. Anal. Chem..

[9] Pollak N., Dölle C. Ziegler (2007). The power to reduce: Pyridine nucleotides—Small molecules with a multitude of functions. Biochem. J..

[10] Murakami K., Yoshino M. (2004). Aluminum decreases the glutathione regeneration by the inhibition of NADP-isocitrate dehydrogenase in mitochondria. J. Cell Biochem..

[11] Sankaran B., Chavan A.J., Haley B.E. (1996). Dentification of adenine binding domain peptides of the NADP^+^ active site within porcine heart NADP^+^-dependent isocitrate dehydrogenase. Biochemistry.

[12] Yoshino M., Yamada Y., Murakami K. (1992). Inhibition by aluminum ion of NAD- and NADP-dependent isocitrate dehydrogenases from yeast. Int. J. Biochem..

[13] Yoshino M., Murakami K. (1992). Aluminum: A pH-dependent inhibitor of NADP-isocitrate dehydrogenase from porcine heart. Biometals.

[14] Wang X., Li K., Yang X.D., Wang L.L., Shen R.F. (2009). Complexation of Al(III) with reduced glutathione in acidic aqueous solutions. J. Inorg. Biochem..

[15] Yang X., Zhang Q., Chen R., Sheng R. (2008). Speciation of aluminum(III) complexes with oxidized glutathione in acidic aqueous solutions. Anal. Sci..

[16] Yang X., Zhang Q., Li L., Shen R. (2007). Flocculation and micellization of sodium dodecyl sulfate solution in the presence of aluminium nitrate: Effect of concentration and temperature. J. Inorg. Biochem..

[17] Yang X., Bi S., Wang X., Liu J., Bai Z. (2003). Multimethod characterization of the interaction of aluminum ion with alpha- ketoglutaric acid in acidic aqueous solutions. Anal. Sci..

[18] Kiss E., Lakatos A., Banyai I., Kiss T. (1998). Interactions of Al(Ⅲ) with phosphorylated amino acids. J. Inorg. Biochem..

[19] Yang X., Bi S., Yang X., Yang L., Hu J., Liu J., Yang Z. (2003). NMR spectra and potentiometry studies of aluminum(III) binding with coenzyme NAD^+^ in acidic aqueous solutions. Anal. Sci..

[20] Karlik S.J., Elgavish G.A., Eichhorn G.L. (1983). Multinuclear NMR studies on Al(III) complexes of ATP and related compounds. J. Am. Chem. Soc..

[21] Atkari K., Kiss T., Bertani R., Martin R.B. (1996). Interactions of aluminum(III) with phosphates. Inorg. Chem..

[22] Ho C.N., Christian G.D., Davidson E.R. (1978). Application of the method of rank annihilation to quantitative analyses of multicomponent fluorescence data from the video fluorometer. Anal. Chem..

[23] Lorber A. (1985). Features of quantifying chemical composition from two-dimensional data array by the rank annihilation factor analysis method. Anal. Chem..

[24] Sanchez E., Kowalski B.R. (1986). Generalized rank annihilation factor analysis. Anal. Chem..

[25] Abdollahi H., Nazari F. (2003). Rank annihilation factor analysis for spectrophotometric study of complex formation equilibria. Anal. Chim. Acta.

[26] Afkhami A., Khajavi F., Khanmohammadi H. (2009). Spectrophotometric determination of complex formation constants between a new Schiff base and some transition metals by rank annihilation factor analysis. J. Chem. Eng. Data.

[27] Frisch M.J., Trucks G.W., Schlegel H.B., Scuseria G.E., Robb M.A., Cheeseman J.R., Montgomery J.A., Vreven T., Kudin N., Burant J.C. (2003). Gaussian 03, Revision B.03.

[28] Markovic J.M.D., Markovic Z.S., Veselinovic D.S., Krstic J.B., Simovic J.D.P. (2009). Study on fisetin-aluminium(III) interaction in aqueous buffered solutions by spectroscopy and molecular modeling. J. Inorg. Biochem..

[29] Allen F.H. (2002). The Cambridge structural database: A quarter of a million crystal structures and rising. Acta Crystallogr. B.

[30] Berman H.M., Westbrook J., Feng Z., Gilliland G., Bhat T.N., Weissig H., Shindyalov I.N., Bourne P.E.  (2000). The protein data bank. Nucl. Acids Res..

[31] Becke A.D. (1993). Density-functional thermochemistry. III. The role of exact exchange. J. Chem. Phys..

